# 478. Contemporaneous Evaluation of Kawasaki Disease and Multi-system Inflammatory Syndrome in Children in Northern Virginia

**DOI:** 10.1093/ofid/ofab466.677

**Published:** 2021-12-04

**Authors:** Andrew Nuibe, Beenish Rubbab, Rebecca E Levorson

**Affiliations:** 1 Pediatric Specialists of Virginia, Fairfax, Virginia; 2 Inova Children’s Hospital, Falls Church, Virginia

## Abstract

**Background:**

Multi-system inflammatory syndrome in children (MIS-C) can present like Kawasaki disease (KD). After Centers for Disease Control and Prevention guidance was issued in May 2020, we implemented local management strategies emphasizing limited laboratory work up of non-toxic children with suspected MIS-C or KD. We then re-evaluated our management recommendations to ensure appropriate resource utilization for children with MIS-C and KD.

**Methods:**

We identified MIS-C and KD cases via convenience sampling of Pediatric Infectious Diseases records at Inova Fairfax Medical Center from May 1, 2020 to February 28, 2021. Manual chart review extracted clinical points of interest and descriptive statistics compared cohorts. Oral changes included edema, erythema, cracking, or strawberry tongue. Abdominal symptoms included pain, emesis, and diarrhea. Respiratory symptoms included shortness of breath, tachypnea, cough, and need for mechanical ventilation. Musculoskeletal symptoms included pain and edema. Neurological symptoms included headache, dizziness, altered mental status, and irritability.

**Results:**

We identified 8 KD cases and 29 concurrent MIS-C cases. MIS-C cases tended to be older and have presenting abdominal symptoms (median age 8 years old versus 2 years old, p < 0.01) and hypotension (20 versus 0, p < 0.01), otherwise there was no difference in the frequency of oral changes, rash, conjunctivitis, musculoskeletal symptoms, or neurological symptoms. 7 KD cases and 8 MIS-C cases did not require intensive care. Patients with MIS-C who did not need intensive care still had a lower initial absolute lymphocyte count (ALC) (median 1275/µL, p < 0.01), lower initial platelet count (median 217/µL, p = 0.05), and higher initial C-reactive protein (CRP) (median 18.3 mg/dL, p = 0.06) compared to KD cases; other results were not different between the two cohorts.

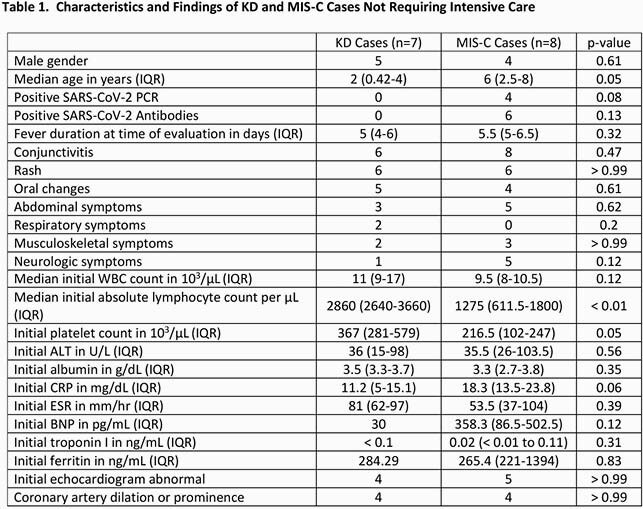

**Conclusion:**

We observed differences in the initial ALC, platelet count, and CRP between KD and MIS-C cases not requiring intensive care, whereas other labs such as ferritin, troponin, B-natriuretic peptide, and initial echocardiograms did not significantly differ between the two cohorts. Thus, our diagnostic management recommending limited laboratory evaluation for non-toxic patients with suspected KD or MIS-C is reasonable.

**Disclosures:**

**All Authors**: No reported disclosures

